# Supplementary material for: Complexome profiling on the Chlamydomonas lpa2 mutant reveals insights into PSII biogenesis and new PSII associated proteins
Source: J Exp Bot. 2021 Aug 26;73(1):245–62. doi: 10.1093/jxb/erab390 (PMC8730698; doi:10.1093/jxb/erab390)
Supplement: erab390_suppl_Supplementary_Dataset_S1 [file erab390_suppl_supplementary_dataset_s1.zip › Supplemental Dataset 1 - Excel List and all profiles/plots/CPLD14_Cre02.g077150.html]

Trivial name: CPLD14  
  
Euclidean distance: 168.37  
Mean Intensity (WT): 37.90  
Mean Intensity (Mut): 33.22  
Distance: 4.44  
  
MapMan:   
  
p value of intensity sums Welch test: 0.9306
